# The roles of ING5 in gliomas: a good marker for tumorigenesis and a potential target for gene therapy

**DOI:** 10.18632/oncotarget.17802

**Published:** 2017-05-11

**Authors:** Shuang Zhao, Zhi-Juan Zhao, Hao-Yu He, Ji-Cheng Wu, Xiao-Qing Ding, Lei Yang, Ning Jia, Zhi-Jie Li, Hua-Chuan Zheng

**Affiliations:** ^1^ Department of Experimental Oncology and Animal Center, Shengjing Hospital of China Medical University, Shenyang 110004, China; ^2^ The First Affiliated Hospital of Jinzhou Medical University, Jinzhou 121001, China

**Keywords:** glioma, ING5, tumorigenesis, chemotherapy, gene therapy

## Abstract

To elucidate the anti-tumor effects and molecular mechanisms of ING5 on glioma cells, we overexpressed it in U87 cells, and examined the phenotypes and their relevant molecules. It was found that ING5 overexpression suppressed proliferation, energy metabolism, migration, invasion, and induced G_2_/M arrest, apoptosis, dedifferentiation, senescence, mesenchymal- epithelial transition and chemoresistance to cisplatin, MG132, paclitaxel and SAHA in U87 cells. There appeared a lower expression of N-cadherin, Twist, Slug, Zeb1, Zeb2, Snail, Ac-H3, Ac-H4, Cdc2, Cdk4 and XIAP, but a higher expression of Claudin 1, Histones 3 and 4, p21, p53, Bax, β-catenin, PI_3_K, Akt, and p-Akt in ING5 transfectants. ING5 overexpression suppressed tumor growth of U87 cells in nude mice by inhibiting proliferation and inducing apoptosis. Down-regulated ING5 expression was closely linked to the tumorigenesis and histogenesis of glioma. These data indicated that ING5 expression might be considered as a good marker for the tumorigenesis and histogenesis of gliomas. It might be employed as a potential target for gene therapy of glioma. PI_3_K/Akt or β-catenin/TCF-4 activation might be positively linked to chemotherapeutic resistance, mediated by ING5.

## INTRODUCTION

Glioma is a most commonly occurring highly malignant primary brain tumor, but its molecular pathways of pathogenesis remain unclear [1]. Although major advancements have been made in surgery, chemoradiotherapy, gene therapy, and immunotherapy of glioma, a poor prognosis remains characteristic of the tumor [2]. The active migration of the glioma cells through the narrow extracellular spaces in the brain makes the glioma cells elusive targets for effective surgical treatment [3].

Since the discovery of class II tumor suppressor ING1 in 1996, five different ING genes (ING1 to ING5) encode the proteins with highly-conserved plant homeodomain motifs, which induces apoptosis, cell arrest, senescence and DNA repair as an important cofactor of p53 [4–6]. Further, they have emerged as a versatile family of growth regulators, phospholipid effectors, histone mark sensors, core components of HDAC1/2 and HAT chromatin-modifying complexes. It has been reported that ING5 inhibit proliferation, cell cycle progression, migration and invasion and epithelial-mesenchymal transition (EMT) of tumor cells [7–9]. ING5 overexprssion was demonstrated to promote glucose catabolism and fat accumulation in lung cancer cells by up-regulating the expression of ADFP, PFK-1, PDPc and HXK1 [10]. Reportedly, ING5 expression was decreased in many malignancies, including breast cancer [11], bladder cancer [12], gastric cancer [13], lung cancer [14], head and neck squamous cell carcinoma [15]. Recently, miR-331-3p and miR-193a-3p might suppress ING5 expression to increase cell proliferation and decrease multi- chemoresistance respectively [12, 16]. Liu et al. [17] found that down-regulated ING5 expression was detected in cells transfected with miR-196a precursor, and accompanied by less apoptosis, higher invasion and proliferation of pancreatic cancer cells.

Here, we observed the effects of ING5 overexpression on the anti-tumor and relevant molecular mechanisms of glioma cells. ING5 overexpression was found to suppress proliferation, energy metabolism, migration, invasion, and induced G_2_ arrest, apoptosis, dedifferentiation, senescence, MET, and chemotherapeutic resistance of U87 cells. We also observed the anti-tumor effects of ING5 in xenograft models of nude mice and clarified the molecular mechanisms. A lower expression of cytoplasmic and nuclear ING5 was for the first time observed in gliomas than normal brain tissues.

## RESULTS

### The effects of ING5 overexpression on biological phenotypes of glioma cells

**Figure 1:**
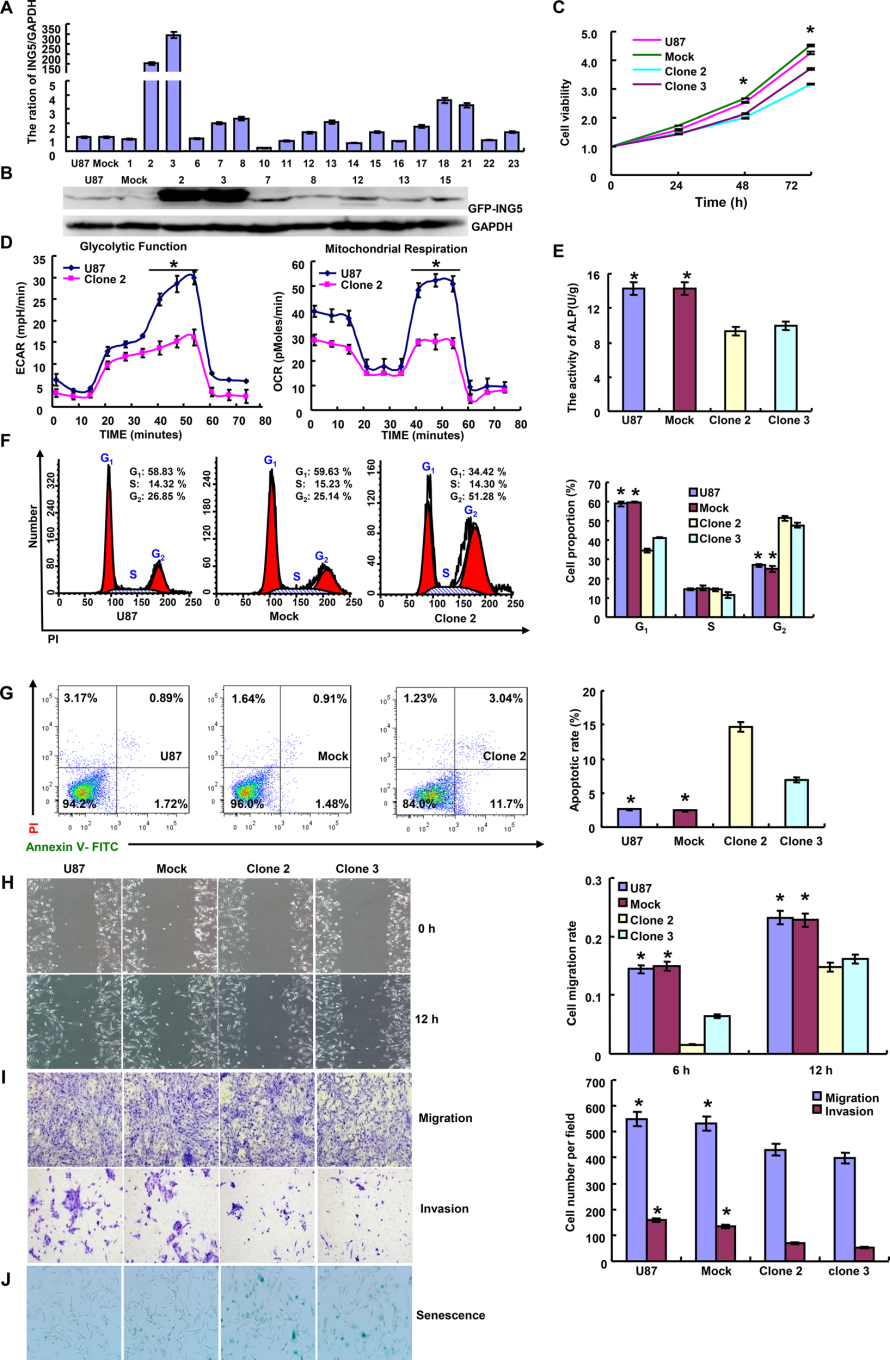
Effect of ING5 on aggressive phenotypes of glioma cells After transfection of pEGFP-N1-ING5, ING5-overexpressing clones were selected by realtime PCR (**A**) and western blot (**B**). ING5 overexpression suppressed proliferation by MTT assay (**C**), glucolysis and mitochondrial respiration by cellular energy metabolism assay (**D**), differentiation by ALP activity (**E**), and induced G_2_/M arrest by PI staining (**F**), apoptosis by Annexin-V staining (**G**) in U87 cells. ING5 decreased the ability of U87 cells to migrate and invade, evidenced by wound healing (**H**) and transwell (**I**) assays. ING5 overexpression also promoted the U87 cell senescence by β-galactosidase staining (**J**), * *p* < 0.05.

**Figure 2:**
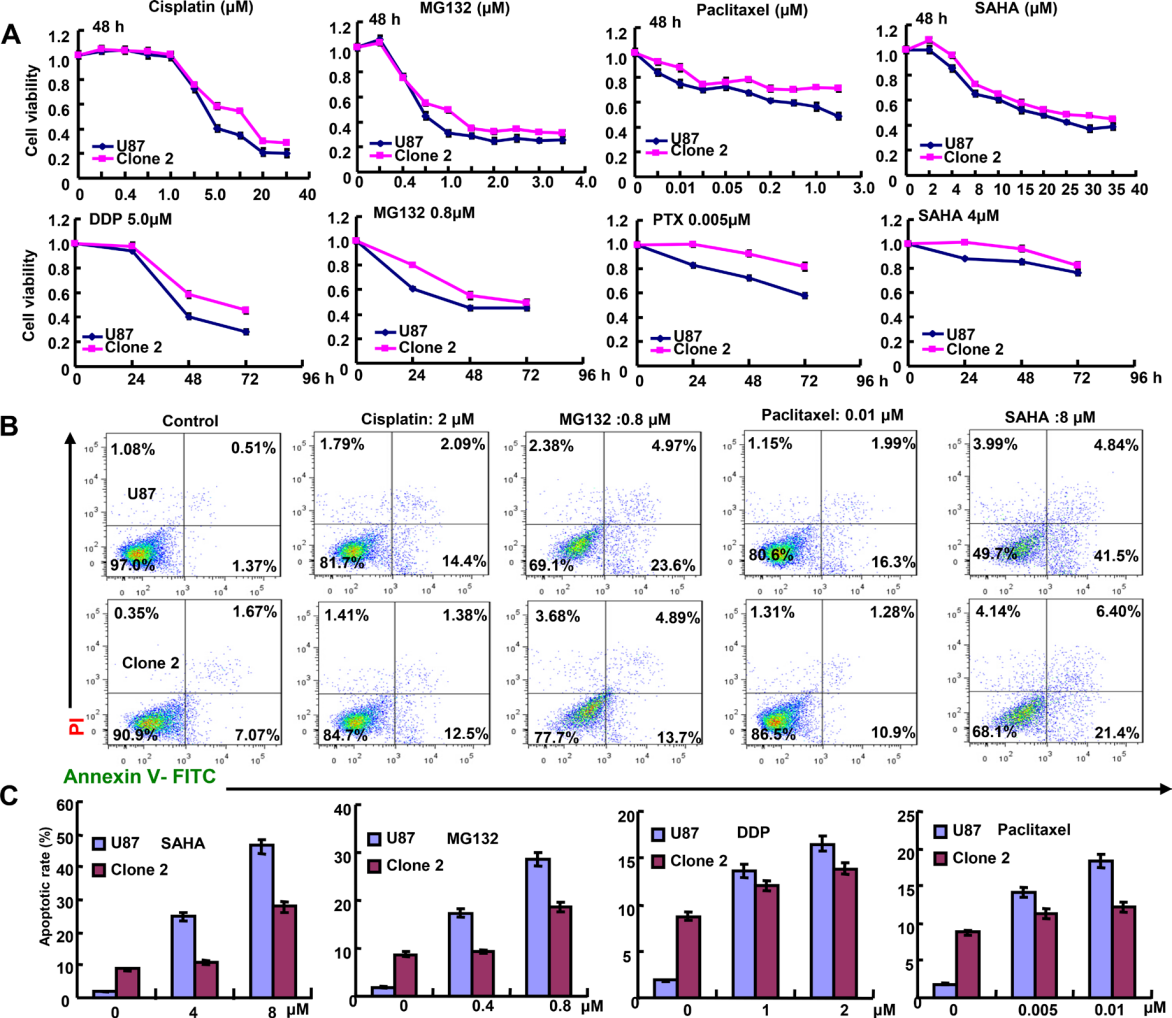
ING5 attenuated the sensitivity of U87 to chemotherapeutic agents After exposed to cisplatin (DDP), MG132, paclitaxel, and SAHA, ING5 transfectants showed a higher proliferation (**A**) and a lower apoptotic level (**B**) than the control in both concentration- and time-dependent manners.

After transfected with pEGFP-N1-ING5, U87 cell showed ING5 overexpression at both mRNA and protein levels (*p* < 0.05, Figure 1A and 1B). ING5 overexpression reduced proliferation, glycolytic function and mitochondrial respiration, and alkaline phosphatase (ALP) activity of glioma cells (*p* < 0.05, Figure 1C–1E). PI staining showed that ING5 overexpression induced G_2_/M arrest and apoptosis in U87 cells (*p* < 0.05, Figure 1F and 1G). The wound healing and transwell assays demonstrated that ING5 decreased cell migration and invasion (*p* < 0.05, Figure 1H and 1I). ING5 also promoted the senescence of U87 cells, evidenced by β-galactosidase staining (Figure 1J). After the exposure to cisplatin, MG132, paclitaxel, and SAHA, U87 transfectants showed higher viability and lower apoptosis than the control in both dose- and time-dependent manners (*p* < 0.05, Figure 2A and 2B).

### The effects of ING5 overexpression on the expression and transcriptional activity of phenotype-related proteins in glioma cells

**Figure 3:**
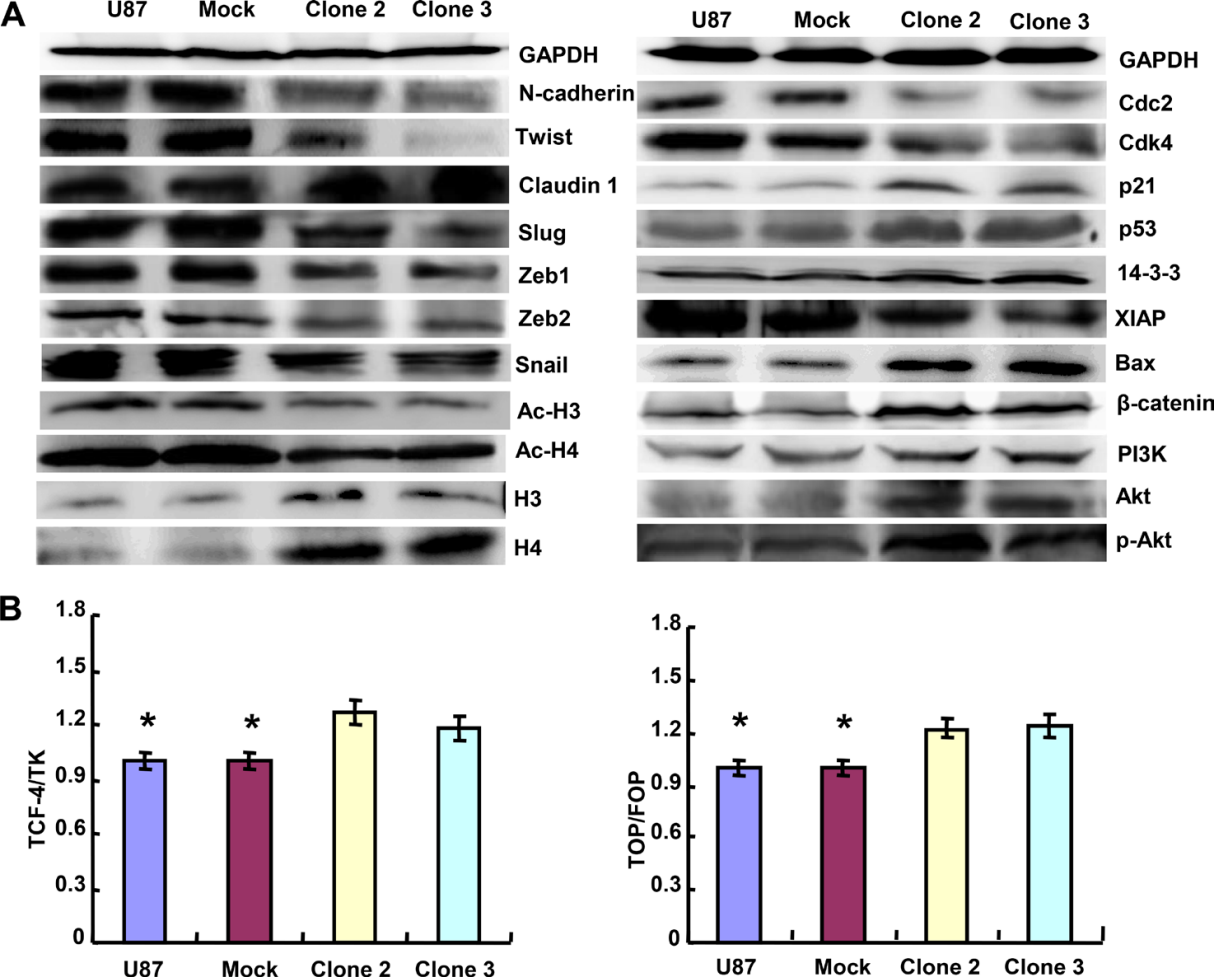
ING5 altered the expression and transcriptional activity of phenotypes-related proteins in U87 cells The phenotype-related molecules were screebed by western blot analysis (**A**). Dual luciferase gene assay demonstrated that ING5 increased TCF-4 promoter activity and TCF-4-mediated gene transcription activity (**B**).*p < 0.05.

As shown in Figure 3A, the protein expression levels of Claudin 1, Histones 3 and 4, p21, p53, Bax, β-catenin, PI_3_K, Akt and p-Akt in ING5 transfectants were higher than those observed in the control and mock cells, while versa for N-cadherin, Twist, Slug, Zeb1, Zeb2, Snail, Ac-H3, Ac-H4, Cdc2, Cdk4 and XIAP. Dual luciferase reporter gene assay demonstrated that both TCF-4 promoter activity and TCF4- mediated gene transcriptional activity became higher in ING5 transfectants than the control and mock cells (*p <* 0.05, Figure 3B).

### ING5 suppressed the tumor growth of glioma cells in xenograft model

**Figure 4:**
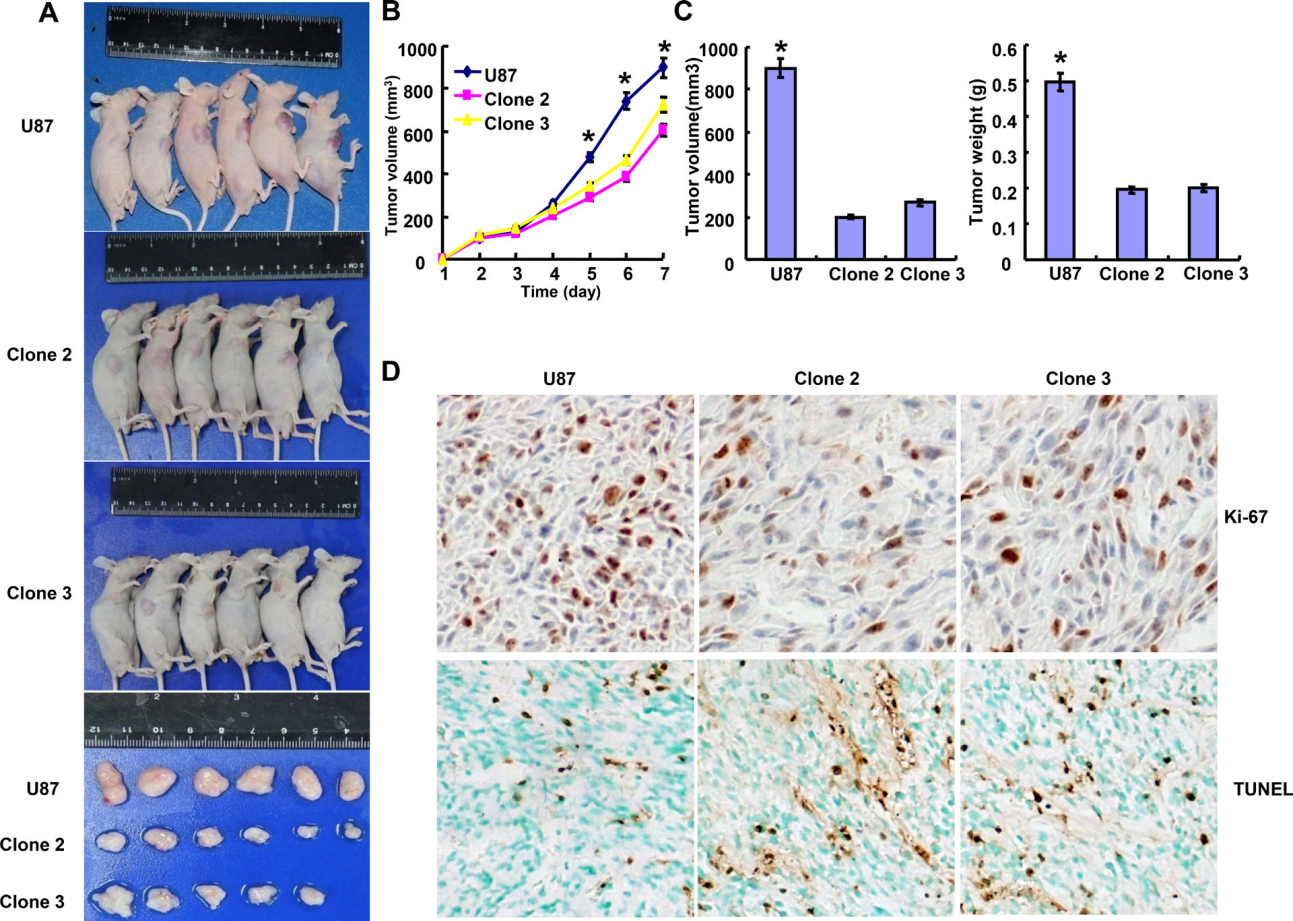
ING5 suppresses the tumor growth of glioma cells *in vivo* The growth of U87 and clone cells were revealed by measurement of tumor size (**A**) and growth curve (**B**) volume and weight (**C**). Immunohistochemistry showed weaker Ki-67 staining and stronger TUNEL staining in clone cells (**D**). *p < 0.05.

As shown in Figure 4A–4C, the tumor volume and weight of ING5 transfectant xenografts become smaller than the control by calculation and weighting (*p* < 0.05). The inhibitory effect was positively correlated with a low proliferation and a high apoptosis in comparison to the control, evidenced by ki-67 immunostaining and TUNEL assay (Figure 4D).

### Relationship between ING5 expression and clinicopathological features of glioma

**Figure 5:**
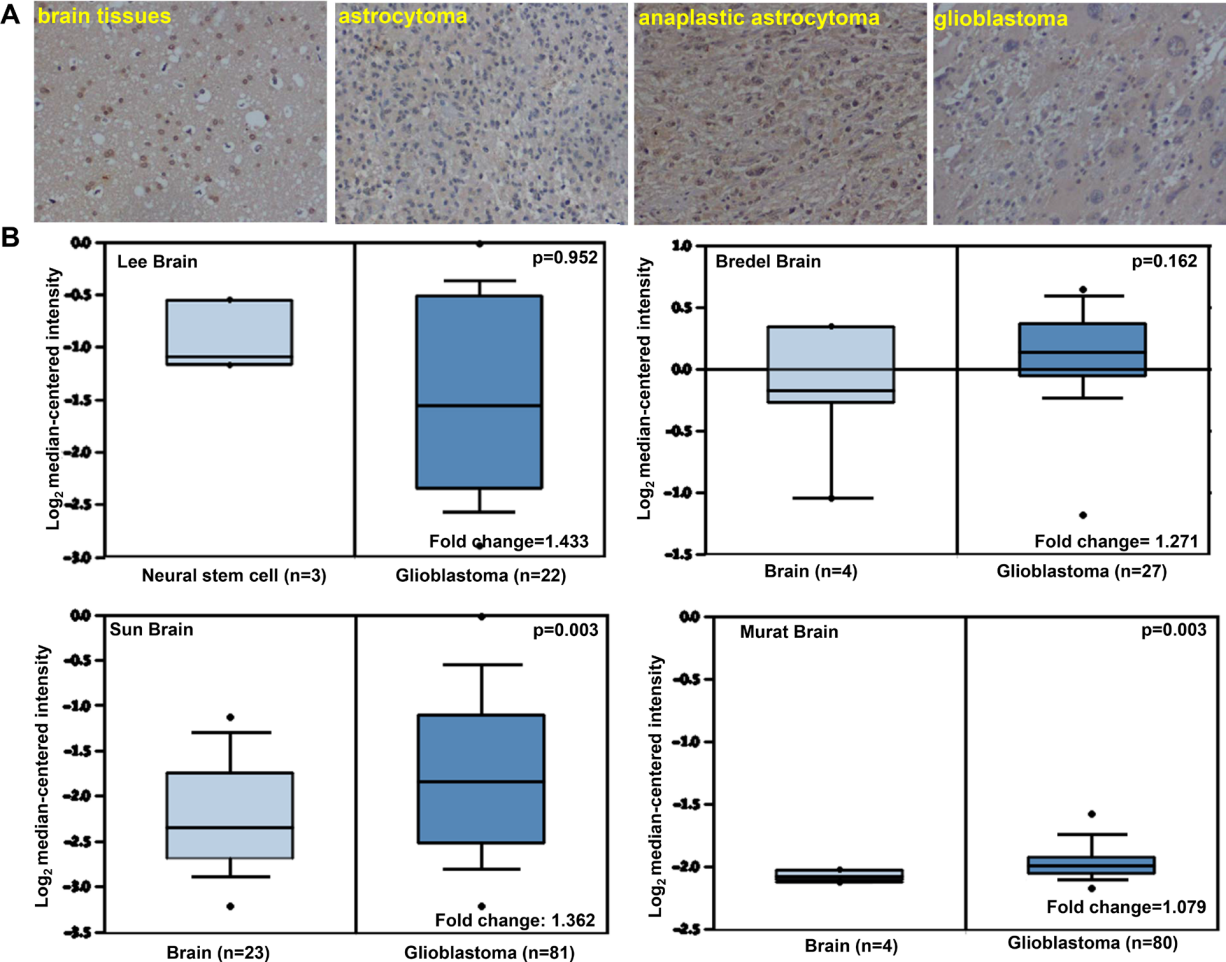
ING5 expression in gliomas According to immunohistochemistry, the positivity to ING5 protein was detectable in the nucleus and cytoplasm of normal brain tissues, astrocytoma, anaplastic astrocytoma and glioblastoma (**A**). Lee’s, Bredel’s, Sun's and Murat's datasets were employed for bioinformatical analysis to compare *ING5* mRNA expression between brain normal tissues and glioblastomas (**B**).

ING5 expression was lower in gliomas than that in normal brain tissue regardless of its subcellular expression pattern (*p* < 0.05, Figure 5A, Table 1). A higher expression of cytoplasmic or nuclear ING5 was detected in anaplastic astrocytoma than astrocytoma and glioblastoma (*p* < 0.05, Table 2). Then, we used Lee’s, Bredel’s, Sun's and Murat's dataset to perform bioinformatics analysis and found that *ING5* mRNA expression was higher in glioblastoma than normal brain tissue from the results of the latter two investigators (*p* < 0.05, Figure 5B). There were no other data for the other subtypes of gliomas.

**Table 1:** ING5 expression in glioma tissues

Groups	*n*	Nuclear ING5 expression						Cytoplasmic ING5 expression					
		-	+	++	+++	PR(%)	*p*	-	+	++	+++	PR(%)	*p*
Normal tissue	56	24	20	7	5	57.1	<0.001	14	19	19	4	75.0	<0.001
Glioma	312	190	32	43	47	39.1		152	73	67	20	51.3	

PR = positive rate.

**Table 2:** Relationship between ING5 expression and the clinicopathological features of gliomas

Clinicopathological features	*n*	Nuclear ING5 expression						Cytoplasmic ING5 expression					
		-	+	++	+++	PR(%)	*p*	-	+	++	+++	PR(%)	*p*
**Sex**													
Male	204	113	16	28	47	44.6	0.601	100	52	38	14	51.0	0.731
Female	156	86	18	26	26	44.9		77	31	38	10	50.6	
**Age/years**													
< 44	161	91	12	20	38	43.5	0.843	78	42	33	8	51.6	0.803
≥ 44	198	108	21	34	35	45.5		99	41	42	16	50.0	
**Grading**													
I–II	150	93	11	16	30	38.0	0.876	72	41	32	5	52.0	0.625
III–IV	118	70	14	20	14	40.7		61	30	21	6	48.3	
**Histological classification**							—						—
Astrocytoma	176	124	10	13	29	29.5		86	50	29	11	51.1	
Anaplastic astrocytoma	42	14	6	11	11	66.7*		15	7	14	6	64.3*	
Glioblastoma	60	33	13	11	3	45.0		35	12	11	2	41.7	

*p < 0.05, compared with astrocytoma and gliblastoma.

## DISCUSSION

*ING5* is localized to human chromosome 2q37.3, contains 8 exons and 7 introns, and encodes 5233 bp cDNA and 28kDa protein of 240 amino acids. ING5 belongs to candidate tumor suppressor, interacts with histone H3K4me3, and promotes the formation of two different HAT complexes to control the histone acetylation [5]. In the present study, lower cytoplasmic and nuclear expression of ING5 in a large number of glioma cases suggested that its down-regulation promoted the tumorigenesis of glioma. ING5 overexpression in anaplastic astrocytoma indicated a close link between ING5 protein and the tumorigenesis of anaplastic atrocytoma. Recently, a low expression of *ING5* mRNA was detectable in oral squamous cell carcinoma [9], but versa from Sun and Burat. The hypoexpression of nuclear ING5 protein and its nucleocytoplasmic translocation were observed in colorectal and gastric cancers, and closely correlated with their aggressive behaviors or adverse prognosis [18, 19].

Reportedly, ING5 overexpression diminished colony-forming efficiency, cell population in S phase, and caused an induction of apoptosis in a p53-dependent manner [20]. In line with the other reports [10, 11, 13, 14], we found that ING5 overexpression inhibited proliferation, glycometabolism, invasion and migration, induced apoptosis, cell cycle arrest, senescence and differentiation of glioma cells. Additionally, ING5 also suppressed the tumor growth of glioma by inhibiting proliferation and inducing apoptosis in tumor-bearing nude mice, in agreement with our *in vitro* data [10, 13]. In combination of these findings, we hypothesized that ING5 might reverse the aggressive phenotypes of glioma cells and be employed as a potential target for gene therapy of glioma.

Li et al. [12] reported that ING5 knockdown increased the chemoresistance and inhibited the DNA damage response pathway in 5637 cells. Mendes-Pereira et al. [21] found that ING5 silencing causes sensitivity to this endocrine agent, tamoxifen in breast cancer cells. Consistent with the results in gastric and lung cancer cells [10, 13], ING5 mediated chemoresistance of glioma cells to various anti-tumor drugs, which paralleled with apoptotic resistance. PI_3_K/Akt /p70S6K pathway is reported to enhance cellular survival and suppress apoptosis because Akt1 inactivates Caspase 9 by phosphorylation on ser196, and disassociates BAD from Bcl-2 or Bcl-xL to lose its pro-apoptotic effect [22, 23]. Wnt signaling pathway can inhibit GSK-3-mediated phosphorylation of β-catenin for the entry of the latter into the nucleus, where the interaction of β-catenin with TCF family transcription factors increases the transcription *c-myc*, *VEGF*, *survivin* and *Cyclin D1* [24]. Here, PI_3_K, Akt, p-Akt and β-catenin were overexpressed, and TCF-4 promoter activity and TCF4-mediated gene transcription activity were increased in ING5 transfectants. Taken together, it was suggested that the activating effects of ING5 on both PI_3_K/Akt and β-catenin/TCF-4 signal pathways contributed to chemotherapeutic resistance in glioma cells.

Both Cyclin D and E activate CDKs and promoted G_1_-S transition, which is inhibited by p21 and p27. Cdc25B activates the cyclin dependent kinase Cdc2 for entry into mitosis [10, 13]. p53 protein can arrest growth by holding the cell cycle at the G_1_/S regulation point and initiate and senescence [25]. Therefore, p21 and p53 overexpression, as well as Cdk4 and Cdc2 hypoexpression would be responsible for the effects of ING5 on the G_2_/M arrest. According to the literatures [26, 27], two truncated fragments of ING5 (aa 1-184 and aa 107-226) can induce cellular senescence and S arrest by down-regulating Cyclin E and Cdk2 expression, while microRNA-193 was found to have pro-proliferation effects for bone mesenchymal stem cells after low-level laser irradiation treatment by targeting ING5 to regulate Cdk2 activity. Bax opens the mitochondrial voltage-dependent anion channel for apoptosis, while XIAP might bind to and suppress Caspase activation [10, 13]. Our investigation indicated that higher Bax expression and lower XIAP expression enhanced apoptosis-inducing events of ING5 via mitochondrial pathway.

Three pathways have been reported to induce EMT, all of which function through three families of transcription factors, including Snail (Snail, Slug), Zeb (Zeb1, Zeb2) and basic Helix Loop Helix (E47, Twist and others) [28]. Claudin 1 is an integral membrane protein and a component of tight junction strands necessary for epithelial sheets [29]. Here, we found that ING5 up-regulated the Claudin 1 expression, but down-regulated the expression of N-cadherin, Slug, Snail, Twist, Zeb1 and Zeb2, indicating that ING5 suppressed the EMT in glioma. Zhao et al. [11] found that ING5 inhibited EMT of breast cancer via PI_3_K/Akt pathway, in agreement with a report about the role of ING5 in lung cancer. It might give a rational explanation for the inhibitory effects of ING5 on migration, invasion and metastasis of cancer cells.

In summary, ING5 overexpression might suppress the proliferation, energy metabolism, migration, invasion, EMT and tumor growth, and induce apoptosis and chemotherapeutic resistance of glioma cells. Down-regulated ING5 expression might be closely linked the tumorigenesis of glioma, and its overexpression with the histogenesis of anaplastic astrocytoma. PI_3_K/Akt or β-catenin/TCF-4 activation might be positively linked to chemotherapeutic resistance, mediated by ING5.

## MATERIALS AND METHODS

### Cell culture

Glioma cells U87 were obtained from the ATCC (Manassas, VA, USA) and grown in a humidified atmosphere of 5% CO_2_ at 37°C in MEM with 10% fetal bovine serum (FBS), 100 U/ml penicillin and 100 μg/ml. Cells were collected and subjected to total RNA and protein extraction. The cells were transfected with pEGFP-N1-ING5, pEGFP-N1 vector or pEGFP-LC3B at 24 h after seeding on dishes, or selected by G418. To check the drug sensitivity, we exposed cells to SAHA, MG132, cisplatin and paclitaxel respectively.

### Proliferation assay

U87 cells and clones were planted at 2.0 × 10^3^ cells per well in 96-well plates and allowed to adhere. At the indicated time points, 20 μl of 5 mg/ml MTT was added to each well, the plates were incubated for 4 h, and then measured at 490 nm.

### Cell cycle analysis

1 × 10^6^ cells were collected, washed by PBS twice and fixed in cold 1 mL 75% ethanol for at least 12 h. Cells were washed twice with PBS again and incubated with 1 ml RNase (0.25 mg/ml) for 1 h at 37°C. Cells were resuspended in propidium iodide (PI) at a concentration of 50 μg/ml and cycle analysis was performed by flow cytometry.

### Apoptosis assay

Flow cytometry was performed with PI and FITC-labeled annexin V (*KeyGEN* Biotech, *China*) following the manufacturer's instructions. In brief, U87 and clone cells were cultured for 48 h, resuspended in 200 μl 1 × Binding Buffer, incubated with 10 μl FITC-Annexin V 15 min in the dark, and resuspended in 300 μl 1×Bingding Buffer. After that, 5 μl PI was added to each tube. Flow cytometry was performed within 1 h and apoptosis analyzed by FlowJo software.

### Alkaline phosphatase (ALP) activity

ALP activity was used as a marker of differentiation. U87 and clone cells were harvested, broken and subjected to the determination of ALP activity and protein concentration using Diagnostics ALP reagent (Sigma, USA) and Biorad protein assay kit (USA). ALP activity was calculated as U per g of protein.

### Cellular energy metabolism

Mitochondrial respiration and glycolysis were measured by Seahorse XF Extracellular Flux Analyzer. Respiration and glycolysis were respectively measured as the rate of oxygen consumption (OCR) and extracellular acidification (ECAR). According to the standard protocol, we injected drugs in the following order: 10 mM glucose, 1.5 μM oligomycin, 50 mM 2-DG for glycolysis assay and 1.5 μM oligomycin, 2 μM FCCP , 0.5 μM Rotenone and antimycin A for mitochondrial respiration. The OCR and ECAR values were determined from 3 wells per sample.

### Wound healing assay

Cell migration was assessed using a wound healing assay as a method described previously [13]. Briefly, cells were seeded at 1 × 10^6^ per well in 6-well culture plates and incubated overnight. A scratch wound was made with a tip, and cultured in FBS-free media, photographed after 6 h and 12 h. The scratch area was measured using image J software.

### Transwell chamber assay

Comparative migration experiments were conducted using a matrigel-coated transwell inserts (BD Bioscience, USA). 2.5 × 10^5^ cells were resuspended in 200 μl serum-free MEM and planted to each insert. 600 μl of media was added to the lower chambers contained 10% FBS. After incubation for 48 h, the cells on the upper surface of the inserts were wiped away with a cotton swab. The cells on the lower surface of the membrane were washed twice with PBS, fixed with 4% paraformaldehyde for 15 min, washed with PBS again, and stained with crystal violet dye (0.1%) for the measurement. For invasive assay, the procedures were the same as above excluding the matrigel coating insert (BD Bioscience).

### β-galactosidase staining

Senescence was determined by senescence-associated β-galactosidase using a senescence cell histochemical staining kit (Beyotime, China). Briefly, U87 and clone cells were firstly fixed for 15 min in paraformaldehyde. After washing with PBS, cells were incubated with β-galactosidase staining solution at 37°C overnight, and then photographed after washing with PBS twice.

### Luciferase reporter assay

Cells were seeded in 24-well dishes and luciferase reporter assay was performed after 48 h of transfection using Dual-Luciferase^®^ Reporter Assay System (Promega, USA). The pGL3-TK was used as a negative control. TCF-4-mediated gene transcription activity was determined by the ratio of pGL3-OT to pGL3-OF luciferase activity, which was normalized to Renilla luciferase activity of the control plasmid, pRL-TK. TCF-4 promoter activity was determined by the value of pGL-[1306] TCF4-Luc luciferase activity, which was also normalized by Renilla luciferase activity of pRL-TK.

### Real-time reverse transcriptase–polymerase chain reaction (real-time RT-PCR)

Total RNA was isolated from glioma cell lines using Trizol (Takara, Kyoto, Japan) and quantified in a Nanodrop spectrophotometer (Wilmington, USA). RT-PCR was performed from 2 μg of total RNA using AMV reverse transcriptase and random primers (Takara). According to the Genbank (NM_032329.4), oligonucleotide primers for *ING5* were designed as follows: forward: 5′ GGGAGATGATTGGCTGTG-3′ and reverse: 5′-CCTTTGGGTTTCGTGGTA-3′ (614-759, 146bp). The primers for the internal control, *GAPDH*, were forward: 5′- CAATGAC CCCTTCATTGACC-3′ and reverse: 5′-TGGAAGATGGTGATGGGATT-3′ (201-335, 135bp). Amplification of cDNA was performed using the SYBR Premix Ex Taq II kit (Takara) using *GAPDH* as an internal control.

### Western blot analysis

Protein assays were performed by Kaumas brilliant blue method. The protein samples were resolved in 12% SDS-PAGE and electrotransferred to a PVDF membrane using standard procedures. The membrane was blocked with 5% skimmed milk in Tris-buffered saline with Tween 20 (TBST) for 1 h and the primary antibody (Table 3) were added on the shaker at 4°C overnight. The membranes were rinsed with TBST, and incubated with anti-rabbit, anti-mouse or anti-goat IgG antibodies conjugated to horseradish peroxidase (HRP; Dako, USA) at a dilution of 1:5000 for 1 h. Bands were visualized with LAS4010 (GE Life Science, USA) by ECL-Plus detection reagents (Santa Cruz, USA).

**Table 3:** Antibodies used for western blot

Name	Source	Company
N-cadherin	Rabbit	Wanleibio
Twist	Rabbit	Wanleibio
Claudin 1 (D-4)	Mouse	Santa Cruz Biotechnology
Slug	Rabbit	Abcam
Zeb1	Rabbit	Wanleibio
Zeb2 (E-11)	Mouse	Santa Cruz Biotechnology
Snail	Rabbit	Wanleibio
Ac-histone 3 (Lys 9/14)	Goat	Santa Cruz Biotechnology
Ac-histone 4 (Lys 8)	Rabbit	Santa Cruz Biotechnology
Histone H3 (N-20)	Goat	Santa Cruz Biotechnology
Histone H4 (H-97)	Rabbit	Santa Cruz Biotechnology
Cdc2 (C-9)	Mouse	Santa Cruz Biotechnology
Cdk4(C-22)	Rabbit	Santa Cruz Biotechnology
p21 (F-5)	Mouse	Santa Cruz Biotechnology
p53	Rabbit	Wanleibio
14-3-3 (H-8)	Mouse	Santa Cruz Biotechnology
XIAP (H-202)	Rabbit	Santa Cruz Biotechnology
Bax (B-9)	Mouse	Santa Cruz Biotechnology
β-catenin	Rabbit	Abcam
PI3K	Rabbit	Abcam
Akt1/2/3 (H-136)	Rabbit	Santa Cruz Biotechnology
p-Akt1/2/3 (Thr 308)	Rabbit	Santa Cruz Biotechnology
ING5	Rabbit	Proteintech
GAPDH	Rabbit	Wanleibio

### Selection of patient samples

A total of 368 glioma cases were collected from surgical resection in The First Affiliated Hospital of Jinzhou Medical University (*n* = 108) between 2002 and 2014, and Shengjing Hospital of China Medical University, Shenyang, China (*n* = 260) between 2007 and 2014. The majority of samples were routinely prepared for storage in pathological blocks. None of the patients had undergone chemotherapy, radiotherapy or adjuvant treatment prior to surgery. Informed written consent was obtained from all participants and the study was approved by the Ethics Committees of both universities.

### Pathology and tissue microarray (TMA) analysis

All tissues were fixed in 10% neutral formalin, embedded in paraffin and sliced into 4 μm-thick sections. The sections were stained with hematoxylin-and-eosin (HE) to for histological analysis. The pathological staging or histological classification was evaluated according to the World Health Organization classification system.

Representative areas of solid tumors were identified in the HE-stained slides of selected tumor samples. Tissue cores were punched out from each donor block and transferred to a recipient block using a Tissue Microarrayer (AZUMAYA KIN-1, Tokyo, Japan). Each recipient block had a maximum of 70 cores. Consecutive 4 μm-thick sections were incised from the recipient block and transferred to poly-lysine-coated glass slides.

### Xenograft models

Female Balb/c nude mice of 6–8 weeks were kept in a specific pathogen-free (SPF) facility with a 12 h light/dark cycle. All animal treatments were in accordance with National Institutes of Health Care and Use of Laboratory Animals. This study was approved by the Institutional Animal Care and Use Committee of our hospital.

U87 and clone cells were grown, detached by trypsinization, followed by washing and re-suspended in serum-free MEM. Subcutaneous xenografts were established by injection of 1 × 10^6^ cancer cells /mouse to the axilla (*n* = 10/group). Tumor growth was then monitored for 7 days and calculated as follows: length × width × width × 0.5. At the end of the experiment, mice from each group was anesthetized, photographed, and sacrificed for further analysis. The volume and weight of xenograft tumor were determined by capacity measurement and weighting. The tumor tissues were fixed in 10% neutral formalin and subjected to the routine block preparation for the following experiments.

### Immunohistochemistry (IHC)

IHC was carried out on 4 μm-thick sections of glioma TMAs were deparaffinized with xylene, rehydrated with alcohol, and subjected to antigen retrieval by heating in target retrieval solution (TRS, Dako) for 20 min in a microwave oven. The sections were quenched with 3% hydrogen peroxide for 5 min to block endogenous peroxidase activity. Non-specific binding was prevented by 5% bovine serum albumin for 5 min.

The sections were incubated with rabbit anti-ING5 (Proteintech, USA) or anti-ki-67 (Dako, USA) for 1 h, and then incubated with anti-rabbit antibodies conjugated to HRP (Dako) for 1 h. After each treatment all sections were washed three times with TBST and the binding sites were visualized with DAB. After counterstaining with Mayer's hematoxylin, the sections were dehydrated, cleared and mounted.

Three independent observers (ZS, ZZJ and ZHC) randomly selected five representative fields from each section. Any discrepancies were checked by both observers until a consensus was reached. Positive expression was graded as follows: – = negative; + = 1%–50%; ++ = 51%–74%; +++ ≥ 75%.

### TUNEL

Terminal deoxynucleotide transferase mediated dUTP nick labeling (TUNEL) was performed using Apoptosis Detection Kit (Millipore, USA). Briefly, the sections were incubated with proteinase K at 37°C for 30 min. Endogenous peroxidase activity was blocked by incubation with 3% hydrogen peroxide in methanol, and then subjected to TUNEL staining. The conjugated horseradish peroxidase was visualized with diaminobenzidine, followed by counterstaining with methyl green.

### Oncomine analysis

The individual gene expression level of *ING5* was analyzed using Oncomine (www.oncomine.org), which a cancer microarray database and web-based data mining platform for a new discovery from genome-wide expression analyses. We compared the differences in *ING5* mRNA level between normal brain tissue and glioblastoma according the data provided by the authors upon request. All data were log-transformed, median centered per array, and standard deviation normalized to one per array.

### Statistical analysis

Statistical evaluation was performed using Spearman's rank correlation to analyze rank data, and Mann-Whitney *U* to differentiate the means of different groups. A *p*-value < 0.05 was considered statistically significant. SPSS v. 10.0 software was employed to analyze all data.
